# Translational Magnetic Resonance Imaging in Autism Spectrum Disorder From the Mouse Model to Human

**DOI:** 10.3389/fnins.2022.872036

**Published:** 2022-05-02

**Authors:** Tomokazu Tsurugizawa

**Affiliations:** ^1^Human Informatics and Interaction Research Institute, National Institute of Advanced Industrial Science and Technology (AIST), Tsukuba, Japan; ^2^Faculty of Engineering, University of Tsukuba, Tsukuba, Japan

**Keywords:** MRI, autism, mouse, translational study, functional connectivity, DTI

## Abstract

Autism spectrum disorder (ASD) is a heterogeneous syndrome characterized by behavioral features such as impaired social communication, repetitive behavior patterns, and a lack of interest in novel objects. A multimodal neuroimaging using magnetic resonance imaging (MRI) in patients with ASD shows highly heterogeneous abnormalities in function and structure in the brain associated with specific behavioral features. To elucidate the mechanism of ASD, several ASD mouse models have been generated, by focusing on some of the ASD risk genes. A specific behavioral feature of an ASD mouse model is caused by an altered gene expression or a modification of a gene product. Using these mouse models, a high field preclinical MRI enables us to non-invasively investigate the neuronal mechanism of the altered brain function associated with the behavior and ASD risk genes. Thus, MRI is a promising translational approach to bridge the gap between mice and humans. This review presents the evidence for multimodal MRI, including functional MRI (fMRI), diffusion tensor imaging (DTI), and volumetric analysis, in ASD mouse models and in patients with ASD and discusses the future directions for the translational study of ASD.

## Introduction

Autism spectrum disorder (ASD) is a heterogeneous syndrome characterized by behavioral features, including deficits in social communication and repetitive behavior patterns, and interests (Hodges et al., 2020). It is currently estimated that 40–80% of patients with ASD have a genetic syndrome and copy number variations (CNVs), with the interaction between environment and genes, likely acting through epigenetic regulation (Rylaarsdam and Guemez-Gamboa, 2019). Magnetic resonance imaging (MRI) is a key tool to non-invasively investigate the functional networks and structural networks in ASD. Patients with ASD show highly heterogeneous abnormalities not only in behavior but also in functional and structural brain connectivity (Salmond et al., 2007; Toal et al., 2010; Chen et al., 2011; Lenroot and Yeung, 2013; Dekhil et al., 2018). The ASD mouse models are useful to investigate the causality between genetic variation and altered brain function in ASD. So far, a lot of ASD mouse models have been generated, yielding dispersed phenotypes that are highly associated with several diagnostic symptoms (e.g., reduced reciprocal social interactions, reduced preference for novel objects, and reduced ultrasonic vocalizations) and dispersed brain function and structure (Silverman et al., 2010; Argyropoulos et al., 2013; Kazdoba et al., 2016b). The high field MRI, which enables the high-resolution functional MRI (fMRI) at the subnucleus level in the mouse brain, will bridge the gap between invasive research in the ASD mouse model and clinical research in patients. This review will compare MRI studies about the ASD mouse models and patients with ASD and will discuss the future direction of translation research on ASD.

## Neuroimaging Technique Using Magnetic Resonance Imaging

**FIGURE 1:**
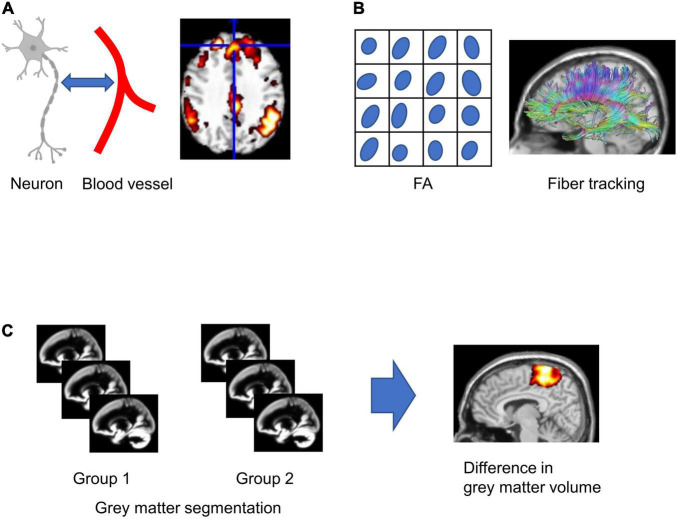
Schematic figures of magnetic resonance imaging (MRI) analysis. **(A)** Functional MRI relies on the neuron-glia vascular interaction. **(B)** Fractional anisotropy (FA) is calculated in each voxel, and fiber tracking is calculated from diffusion tensor imaging (DTI). **(C)** For voxel-based morphometry (VBM), gray matter segmentation in each subject was performed, and the statistical difference in gray matter concentration between groups in each voxel was analyzed.

The attractive point of MRI is the non-invasive multimodal imaging of the structure and function of the brain (Hao et al., 2013; Figure 1). The fMRI is a neuroimaging technique using blood oxygen level-dependent (BOLD) signals that rely on the fluctuation of the blood deoxyhemoglobin content in the vessels associated with neuronal activation (Ogawa et al., 1990; Figure 1A). Task-based fMRI is performed with the passive sensory stimulation (e.g., visual or auditory stimulation) or the active task (e.g., decision-making or cognitive task) to investigate the neuronal activation during a specific task. In contrast, functional connectivity, which reflects the synchronization of fluctuation of BOLD signals accompanying neuronal activity among anatomically separated brain regions, is calculated using the resting-state fMRI without stimuli or tasks (Friston, 2011). Depending on the method of resting-state analysis, functional connectivity analysis can indirectly indicate several neural networks, which are strongly functionally connected at rest and are related to cognitive function (Li et al., 2017). Diffusion tensor imaging (DTI) detects the displacement of a water molecule in white matter (Le Bihan et al., 2001; Kumamoto and Tsurugizawa, 2021) and can indirectly measure the degree of anisotropy and the structural orientation of projecting fiber (Basser et al., 1994; Figure 1B). DTI estimates the mean diffusivity (MD) and fractional anisotropy (FA), indicating the degree of water diffusion in the tissue and degree of anisotropy of the fiber projection, respectively (Peeters et al., 2001). In addition to these indicative values, DTI tractography visualizes white matter fiber structure (Figure 1B; Basser et al., 2000). Voxel-based morphometry (VBM) is an automated method that calculates voxel-by-voxel gray matter density from structural T1- or T2-weighted images and allows voxel-wise comparison of local gray matter density between groups (Whitwell, 2009; Figure 1C). The MRI multimodal imaging shows the change in function and structure in psychiatric diseases and neurodevelopmental diseases (Lee et al., 2005; Abhinav et al., 2014; Woodward and Cascio, 2015; Lui et al., 2016). When we perform the comparative study between mouse model imaging and clinical imaging, we should consider the different sizes of the brain. As the size of the mouse brain is around 3,000 times smaller than the human brain (Hofman, 2014), a higher resolution is required for the mouse brain rather than that for humans. For example, the resolution of fMRI in humans with 3T MRI is typically 2–3 mm iso-voxel, while the resolution in mice requires at least in-plane resolution of 200–300 μm in slices of 0.5–0.8 mm thick. To achieve this resolution, a high magnetic field and dedicated radio frequency (RF) coil for a small mouse brain are essential. A high field magnet greater than 7T with a multichannel RF coil is useful with high resolution and high-quality images to investigate the rodent brain (Abe et al., 2019; Boido et al., 2019; Tsurugizawa et al., 2019; Wang et al., 2020).

Besides the structural and functional MRI studies, molecular imaging using positron emission tomography (PET) and single-photon emission computed tomography (SPECT) are useful tools to investigate the neurophysiological mechanisms in patients with ASD (Hwang et al., 2017). This molecular imaging is also essential to compare the neurotransmitter release in ASD mouse models and patients with ASD directly.

## Autism Spectrum Disorder Mouse Model

Table 1 shows the MRI studies using genetically modified ASD mouse models with the target of ASD risk gene. The recombinant of the target gene, which abnormally exists or lacks in patients with ASD, has been performed in the mice. In addition to the behavioral phenotype (Burrows et al., 2015; Thompson and Levitt, 2015; Kazdoba et al., 2016a; Martin et al., 2018; Saito et al., 2021), functional and structural abnormalities in ASD mouse models have been investigated.

**TABLE 1:** ASD mouse models and MRI studies.

ASD mouse model/risk genes	Resting state/Task-based fMRI	DTI/Volumetry	Phenotype	Abnormal brain function and structure	References
15q11-13 (dup)	Y/Y	Y/Y	Reduced, ultrasonic vocalization, social interaction	Widespread hypo-functional and structural connectivity	Takumi, 2011; Ellegood et al., 2015b; Tsurugizawa et al., 2020
16p11.2 (±)(df/ +)	Y/N	N/Y	Reduced sociability	Impaired functional connectivity in the prefrontal cortex and structure in the basal ganglia	Portmann et al., 2014; Bertero et al., 2018; Zerbi et al., 2021
22q11.2 (df(16)A ±)	N/N	N/Y	Impaired social recognition	Volumetric alteration in the cortico-cerebellar, the cortico-striatal and the cortico-limbic circuits	Ellegood et al., 2014; Saito et al., 2021
FMR1 (−/y)	Y/N	N/Y	Impaired sensorimotor gating, seizure susceptibility, and repetitive behavior	Structural hyperconnectivity in the V1 and low connectivity between the V1 and other neocortical regions	Ellegood et al., 2010; Haberl et al., 2015; Pacey et al., 2013
Mecp2 (−/y)	Y/N	N/Y	Respiration deficits	Hypo-functional Connectivity in the insula, the sensorimotor cortex, caudate putamen, and the hippocampus	Ward et al., 2008; Nag et al., 2009; Zerbi et al., 2021
XO	N/N	N/Y	Anxiogenic	Volume decreases in the olfactory bulb, and thalamic nuclei	Isles et al., 2004; Raznahan et al., 2013
Gtf2i (dp/dp)(±)	N/N	N/Y	Overly social behavior	Mixture of increases and decreases of the volumes in whole brain	Ellegood et al., 2015a; Martin et al., 2018
Shank3 (±)	Y/N	N/Y	Increased repetitive self-grooming and impaired novel object recognition	Hypo-functional connectivity in the limbic system and the insular cortex. Increases in many of the white-matter structures	Ellegood et al., 2015a; Kazdoba et al., 2016a; Schoen et al., 2019; Zerbi et al., 2021
Nl3 (−/y)	N/N	N/Y	Abnormal aggression Increased repetitive behavior	Thinning corpus callosum, and altered volume of gray matter in the hippocampus, the striatum, and the thalamus	Radyushkin et al., 2009; Ellegood et al., 2011; Burrows et al., 2015
Met-Emx1	Y/N	N/Y	Hypoactivity	Impaired large-scale somatosensory network connectivity. Large volume in the rostral cortex, caudal hippocampus, and the thalamus.	Smith et al., 2012; Thompson and Levitt, 2015; Tang et al., 2019
Itgβ3 (−/−)	N/N	N/Y	Increased grooming behavior	Decreased cerebellum volume	Ellegood et al., 2012; Steadman et al., 2014
Engrailed2 (En2)	Y/N	N/Y	Reduced social sniffing, allogrooming, and less aggressive behavior	Reduced functional connectivity in the somatosensory cortex. Large white matter structures	Ellegood et al., 2015a; Chelini et al., 2019
BALB/cJ	N/N	N/Y	Reduced social interaction	Decreased FA in the somatosensory cortex and the external capsule	Kumar et al., 2012
BTBR	Y/N	N/Y	Increased repetitive behaviors	Reduced cortico-thalamic function and increased activity in the hypothalamus and the dorsal hippocampus. Disconnectivity in the fronto-thalamus and striatum	Dodero et al., 2013; Ellegood et al., 2013; Sforazzini et al., 2016

### Functional Connectivity Studies in Autism Spectrum Disorder Model Rodents

Depending on the ASD mouse model, the resting-state fMRI indicates the hypo-connectivity in a specific network or global network. A mouse model for human 15q11-13 duplication (*15q dup*) is the first CNV model of ASD (Nakatani et al., 2009). It is reported that adult *15q dup* mice display abnormal behavior, including poor social interaction, behavioral inflexibility, abnormal ultrasonic vocalizations, and correlates of anxiety (Nakatani et al., 2009; Tamada et al., 2010). Adult *15q dup* mice show widespread functional hypo-connectivity in the brain (Tsurugizawa et al., 2020). Chromosome 16p11.2 deletion is also one of the most common CNVs in autism (Walsh and Bracken, 2011). Autism-associated 16p11.2 microdeletion in adult mice impairs prefrontal functional connectivity and weak long-range functional coupling with temporal-parietal regions (Bertero et al., 2018). The Fragile X Mental Retardation gene (FMR1) knockout mice exhibit some of the physical and behavioral characteristics of human fragile X syndrome (Kazdoba et al., 2014). The FMR1 knockout mice show abnormalities in the myelination of cerebellar axons in the first postnatal week, indicating delayed myelination in the FMR1 mouse brain (Pacey et al., 2013). The hypo-connectivity between the hippocampus and the neocortical areas, as well as intracortical connectivity, is found in FMR1 knockout mice (Haberl et al., 2015). Engrailed-2 (En2) knockout adult mice, which show a lower expression of FMR1 and anatomical defects common to FMR1 knockout, significantly reduce synchronization in the somatosensory-auditory/associative cortices and the dorsal thalamus (Chelini et al., 2019). Functional connectivity between the somatosensory cortex and the thalamus is significantly reduced in conditionally mutant adult Met-Emx1 mice expressing a kinase-dead Met restricted to the cerebral cortex and the hippocampal structures (Tang et al., 2019). Adult BTBR T + Itpr3tf/J (BTBR) mice, originally bred for studies on insulin resistance, diabetes-induced nephropathy, and phenylketonuria, show a reduction in long-range functional connectivity involving retrosplenial areas and lateral cortical regions (Sforazzini et al., 2016). Recently, Zerbi et al. classified 4 abnormal functional connectivity subtypes from 16 types of ASD mouse models (Zerbi et al., 2021). Across all 4 clusters, the vulnerability to abnormal connectivity was observed in the sensorimotor regions, the olfactory and the cortical subplate, the pallidum, and the hypothalamus. Because etiological variability is a key determinant of connectivity heterogeneity in ASD, identification of etiologically relevant connectivity subtypes may advance the diagnosis of ASD.

### Task-Based Functional MRI Studies in Autism Spectrum Disorder Mouse Model

Compared with the resting-state functional connectivity, task-based fMRI has rarely been performed in the ASD mouse model. This is because, in addition to the limited space in MRI bore, mouse fMRI has been performed with light anesthesia. Anesthesia reduces consciousness, and therefore, cognitive tasks cannot be performed under anesthesia, even if residual brain function at the resting state is equivalent to an awake state (Tsurugizawa and Yoshimaru, 2021). This limits the opportunities for a comprehensive understanding of brain function. To overcome this limitation, we constructed awake fMRI with an odor delivery system (Tsurugizawa et al., 2013, 2020). The stranger-odor was chosen because the olfaction is essential for interpersonal perception in the mouse, and the ASD mouse model shows a deficit of reciprocal social interaction. BOLD response to odor stimulation of stranger mouse was observed in the locus coeruleus/lateral parabrachial nucleus, the thalamus, the hypothalamus, and the hippocampus in addition to the olfactory bulb in adult control mice. While the BOLD response in the olfactory bulb to odors of stranger mice was observed in adult *15q dup* mice, those in the other regions were suppressed. This result indicates that *15q dup* mice show abnormalities in the processing of the odorant information rather than basic odor recognition and olfactory sensing functions supported by the olfactory bulb. Both abnormal BOLD response and poor social interaction in *15q dup* mouse were rescued by acute D-cycloserine injection, which is a partial agonist of NMDA receptors and may be effective in ameliorating autistic-like behavior of patients with ASD (Minshawi et al., 2016; Wink et al., 2017; Tsurugizawa et al., 2020). This indicates that recovered activation to stranger-odor presentation could be related to behavioral recovery. Resting-state fMRI is used to investigate the functional segregation of brain regions/networks for a better understanding of the organization and origination of the brain’s cognitive functioning, while task-based fMRI is widely adopted to identify brain regions that are functionally involved in specific task performance (Di et al., 2013; Cole et al., 2021). In addition to *15q dup* mice, task-based fMRI in other ASD mouse models is required for the comprehensive understanding of the altered brain function associated with ASD risk genes in a future study.

### Structural Studies in Autism Spectrum Disorder Mouse Model

The structural MRI reflect the biological alterations of microscopic structure in ASD mouse models (Lerch et al., 2017). DTI has a unique ability to delineate axonal tracts within the white matter. This has not been possible with other non-invasive imaging techniques. The structural studies in the ASD mouse models report the abnormality of white matter structure in adult ASD compared with control. The widespread reduction of white matter fibers is found in *15q dup* mice (Tsurugizawa et al., 2020). This widespread reduction would correspond to the widespread functional hypo-connectivity as described above. A DTI study in BALB/cJ mice, exerting low levels of social interaction, reveals a significant difference in the growth trajectory of the FA from the external capsule in comparison with control between P30 (postnatal day) and P50, but not at P70 (Kumar et al., 2012). The white matter structure and the regional volume in the somatosensory cortex and the thalamic nuclei were significantly decreased in adult BTBR/cJ mice compared with the control (Ellegood et al., 2013). Shank3 transgenic mice had smaller thalamus (Schoen et al., 2019). Tbx1, a gene encoded in 22q11.2 CNV heterozygous mice, shows a significant decrease in FA, and the microscopic analyses show that Tbx1 heterozygous mice exhibited an apparent absence of large myelinated axons and thicker myelin in medium axons in the fimbria, resulting in an overall decrease in myelin. This importantly indicates that the altered FA in the ASD animal model is likely to be related to the altered neuroanatomical structure (Hiramoto et al., 2021).

Voxel-based morphometry and volumetric analysis in the ASD mouse model reveal volume increase or decrease in specific regions depending on the ASD risk genes, but some of the regions, such as the cerebellum and the thalamus, are commonly decreased. The *15q dup* mice decrease the gray matter volume in the dentate gyrus, the medial striatum, and the dorsal raphe nucleus, which may be the cause of the reported serotonin defects (Ellegood et al., 2015b). Adult 16p11 ± male mice, which increase the circling behavior and show normal social behavior, increased volume in the globus pallidus (Portmann et al., 2014). Adult mice with recurrent deletions at the 22q11.2 locus (Df(16)A ±) show smaller volume in the cerebellum rather than the wild type (Ellegood et al., 2014). Bilateral hemispheric areas including the flocculus and the para-flocculus, the crus I dorsal surface, and medial aspects of the anterior lobule are affected. Within the vermis, the lobules IV/V, IX, and X show a robust volume decrease in the adult Df(16)A ± mice compared with the wild-type mice. In adolescent FMR1 knockout mice, a significant volume decrease is found in the nucleus interpositus and the fastigial nucleus of the cerebellum (Ellegood et al., 2010). Adolescent mice with no expression of functional methyl CpG binding protein 2 (MeCP2) protein decrease the whole brain volume (Ward et al., 2008). Additionally, cerebellar and ventricular volumes are also decreased in male MeCP2 mutant mice (Nag et al., 2009). Adult murine X-monosomy (XO) mice show an abnormal volume decrease in the olfactory bulb, the thalamus, and the hypothalamus. The volume of the frontal and the parietal cortices is increased compared with XX and XY mice (Raznahan et al., 2013). Adult Neuroligin3 R451C knockin (NL3 KI) mice decrease the total brain volume (Radyushkin et al., 2009) and gray matter volume in the hippocampus, the striatum, and the thalamus (Ellegood et al., 2011). Adult Met-Emx1 mice increase the rostral cortex, the caudal hippocampus, the dorsal striatum, the thalamus, and the corpus callosum relative to the control mice at P50 and P70, but not at P30 (Smith et al., 2012). Adult integrinβ3 (ITGβ3) knockout mice decrease the total brain volume (Ellegood et al., 2012). Ellegood et al. compared the volume change among twenty-six adult ASD mouse models (Ellegood et al., 2015a). Overall, the volume decrease in the parieto-temporal lobe, the cerebellar cortex, the frontal lobe, the hypothalamus, and the striatum is consistently found in all ASD mouse models. The clustering of these mice produces three large groups (Ellegood et al., 2015a). These results indicate that the variation of the volume change in the ASD mouse model has common abnormal volume change depending on the type of the etiology of the ASD risk gene.

## Clinical Studies

Compared with mouse MRI, clinical MRI has investigated the function and the structure profoundly from toddlers to adults. In most MRI studies, the subjects with ASD have similar IQ (generally > 80) with typically developing (TD) subjects to compare the functional and structural networks between ASD and TD.

### Resting-State Functional Studies in Patients With Autism Spectrum Disorder

Resting-state fMRI studies in patients with ASD indicate age-dependent alteration of functional connectivity. Patients with ASD show broad functional hyper-connectivity (over-connectivity) during development and broad functional hypo-connectivity (under-connectivity) in adults (Supekar et al., 2013; Ha et al., 2015; Haghighat et al., 2021). The subregions of the insular cortex, such as the left ventral agranular insula, the right ventral dysgranular, the granular insula, and the dorsal dysgranular insula, are involved in hyper- and hypo-connectivity in children with ASD compared with TD children (Xu et al., 2018). Default mode network (DMN), which is a large-scale brain network and is a brain system for processing information about the “self” and “other” (Mars et al., 2012), is disrupted in both children and adults with ASD compared with TD (Yerys et al., 2015; Padmanabhan et al., 2017). The intrinsic functional connectivity studies in children with ASD are also found to increase within-network connectivity between core DMN nodes (Lynch et al., 2013), while studies in ASD adolescents and adults report decreased connectivity (Cherkassky et al., 2006; Kennedy and Courchesne, 2008). In addition to DMN, increased intrinsic functional connectivity between visual and sensorimotor networks is found in young children with ASD compared with TD controls (Chen et al., 2021). Age-related reduction of the visual-auditory between-network connectivity is also reported in the ASD group but not in the TD group (Chen et al., 2021). Functional connectivity between the left dentate nucleus in the cerebellum and the cerebral regions involved in DMN is decreased in adults with ASD compared with TD adults, while predominant cerebro-cerebellar functional connectivity is increased in the ASD group (Olivito et al., 2017).

Some studies report local hyper-connectivity in the frontal, temporal and occipital lobes in children and adolescents with ASD (Keown et al., 2013; Maximo et al., 2013); other studies do not detect any local hyper-connectivity in children and adolescents with ASD (von dem Hagen et al., 2013; Bernas et al., 2018; Wu et al., 2021). These discrepancies in the results of ASD fMRI can be attributed to small sample sizes, phenotypic heterogeneity among subjects with ASD, and the use of different analytical methods across studies (Zhang et al., 2016). A meta-analysis of ASD resting-state fMRI, including 424 subjects, reveals the local hypo-connectivity in the dorsal posterior cingulate cortex and the right medial paracentral lobule (Lau et al., 2019). A meta-analysis with a large sample size will contribute to uncovering a consistent pattern of resting-state local abnormalities that may serve as potential neurobiological markers for ASD.

### Task-Based Functional Magnetic Resonance Imaging to Investigate the Integrative Sensory Processing in Patients With Autism Spectrum Disorder

Autism spectrum disorder is characterized by sensory-based behavior and difficulty with sensory integration. Particularly, impaired face processing is a characteristic in patients with ASD. Previous fMRI studies summarize the abnormality of sensory processing, face recognition, and multi-sensory integration (Bird et al., 2006; Marco et al., 2011; Weigelt et al., 2012; Nomi and Uddin, 2015; Ocak et al., 2018). The fMRI with the face recognition task shows weak or no activation in the fusiform gyrus in adults with ASD and significantly reduced activation in the inferior occipital gyrus, superior temporal sulcus, and amygdala compared with TD adults (Pierce et al., 2001). Both children with ASD and TD children activate a right premotor/prefrontal system when identifying images containing a greater percentage of the self-face (Uddin et al., 2008). TD children show activation of this system during both self-face and other-face processing, but children with ASD only activate this system mostly during self-face processing (Uddin et al., 2008). The repeated presentation of fearful faces induces the habituation of BOLD response in the left amygdala in TD adults. In contrast, habituation of BOLD response in the left amygdala in adults with ASD is significantly smaller than that in TD adults (Kleinhans et al., 2016). Individuals with ASD and TD controls show similar BOLD responses in the fusiform face area during a face-processing task, in which they are asked to match faces presented in the upright versus inverted position, with both groups showing increased BOLD response to inverted faces (Bookheimer et al., 2008). However, it is only in the TD group that BOLD responses are found in several brain regions involved in the prefrontal cortex and amygdala (Bookheimer et al., 2008). These results indicate that frontal and amygdala dysfunction is associated with impaired face processing in ASD.

Auditory distractors affect the neural processing of emotion identification, and ASD shows different brain responses to auditory stimulation. Compared with TD children and TD adolescents, the right temporal cortex response to the sound is greater in children and adolescents with ASD (Jou et al., 2010). This difference can be explained by the increased volume of right hemisphere superior temporal gyrus volume in ASD (Jou et al., 2010). The unbalanced response to the language sounds in the right superior temporal gyrus is also observed in ASD toddlers (12–48 months; Eyler et al., 2012). Adolescents with ASD show a greater increase of BOLD response in the right amygdala, the right putamen, the hippocampus, the insula, and several temporal and occipital regions when stimulated by aversive environmental sounds (e.g., lawnmowers and police sirens) rather than TD controls (Patterson et al., 2021). Habituation to repeated audio stimulation in adults with ASD has also been assessed using fMRI in the previous study (Millin et al., 2018). The post-transient sustained BOLD response in the auditory cortex is greater in individuals with ASD than TD controls when habituation to the sound is evoked by fixed interval timing conditions. Together with the face-processing study, adults with ASD are characterized by reduced habituation of the face and auditory processing to the repeated face and auditory presentation.

Integration of sensory information is also described in previous studies (Stevenson et al., 2014a,b; Baum et al., 2015; Foxe et al., 2015; Hornix et al., 2019). The reviews highlight the importance of multisensory processing in building perceptual and cognitive representations, and deficits in multisensory integration may also be a core feature of ASD. Murray et al. revealed the significant difference in BOLD response between adults with ASD and TD controls during visual, auditory, and motor stimuli (Murray et al., 2020). The BOLD response in the middle-temporal sulcus by these multiple stimuli is increased and sustained in adults with ASD compared with TD controls (Murray et al., 2020). In contrast, Stickel et al. reported similar networks, including the primary visual cortex, the inferior parietal sulcus, and the medial/inferior frontal cortices, during olfactory-visual and auditory-visual integration processes between adults with ASD and TD adults (Stickel et al., 2019). BOLD signal in the amygdala is increased specifically during olfactory-visual integration, and the BOLD signal in a superior temporal is increased during auditory-visual integration in both groups. The left auditory cortex (BA41) shows stronger activation in TD controls than in subjects with Asperger’s syndrome (Tietze et al., 2019).

### Structural Changes in Patients With Autism Spectrum Disorder

Diffusion tensor imaging is an essential tool to investigate the abnormalities in white matter structure in individuals with ASD. Travers et al. summarized forty-eight DTI types of research in patients with ASD (Travers et al., 2012). White matter integrity spanning across many regions of the brain is decreased in individuals with ASD, and the regions such as the corpus callosum, the cingulum bundles, and white matter tracts that pass through the temporal lobe are most consistently decreased (Travers et al., 2012). Young ASD toddlers have a higher tract FA and volume than TD toddlers, but by 3 to 4 years of age, this increase in FA and volume is disappeared (Solso et al., 2016). Additionally, widespread reduction in FA and increased MD are reported in adults with ASD compared with TD controls (Gibbard et al., 2013). For DTI analysis, careful quality control and head motion-matching should be considered in DTI with ASD because head motion is more dominant in the quality of DTI (Tijssen et al., 2009). To assess the effect of head motion on FA in ASD, Solders et al. subdivided the adults with ASD into three groups and assessed the effect of the motion in each group: full sample (FS), quality controlled (QC), and quantitatively matched (QM). In the FS group, FA was reduced in the adults with ASD compared with the TD group throughout the right hemisphere. This effect was less extensive in the QC group and absent in the QM group (Solders et al., 2017). These results indicate that the DTI data in ASD should be treated carefully and require quality control and motion-matching.

The altered gray matter volume in ASD can be investigated using VBM. The gray matter volume in the middle temporal gyrus, the fusiform gyrus, the amygdala, the middle frontal gyrus, and the medial superior frontal gyrus is reduced in adults with ASD compared with those in TD controls (Sato et al., 2017). The volume in the amygdala is increased in children with ASD but this structural change is independent of non-verbal IQ (Sparks et al., 2002). In contrast, in children with ASD, decreases are observed within fronto-striatal and parietal networks, and additionally in the ventral and the superior temporal gray matter (McAlonan et al., 2005). Furthermore, gray matter volume in cerebellar lobule VII is decreased in children with ASD (D’Mello et al., 2015). Interestingly, VBM analysis using Autism Brain Imaging Database Exchange (ABIDE), which contains over 1,100 participants, reveals that total brain and gray matter volumes are greater by approximately 1–2% in individuals with ASD than in TD controls (Riddle et al., 2017). Additionally, the left anterior superior temporal gyrus is larger in individuals with ASD than in TD controls. However, there is no significant reduction in the correlation between IQ and total brain volume in ASD compared with TD.

### Molecular Imaging in Patients With Autism Spectrum Disorder

Several studies use PET and SPECT in individuals with ASD to investigate the altered binding activity of neurotransmitter receptors. The serotonin transporter binding in the thalamic nuclei is significantly lower throughout the brain in adult individuals with ASD compared with controls, while dopaminergic binding in the orbitofrontal cortex is significantly increased in adult individuals with ASD (Nakamura et al., 2010; Beversdorf et al., 2012). The SPECT with [123I] nor-β-CIT study reveals that serotonin transporter binding capacity is reduced in the medial frontal cortex, the midbrain, and the temporal lobe areas (Makkonen et al., 2008). Importantly, the abnormality in the serotonergic system is also observed in the ASD mouse models (Takumi, 2011; Guo and Commons, 2017). The metabotropic glutamate receptor 5 (mGluR5) in cerebellar vermis is elevated in individuals with ASD (Fatemi and Folsom, 2011). The binding of mGluR5 tracer [18F]-3-fluoro-5-[(pyridin-3-yl)ethynyl]benzonitrile ([18F]-FPEB) is increased in the cerebellum (Fatemi et al., 2018). The GABAA α5 subunit binding is reduced throughout the brain of 3 adult men with ASD compared with controls (Mendez et al., 2013). However, Horder et al. reported no differences in GABA_A_ receptor or GABA_A_ α5 subunit availability in any brain region of adults with ASD compared with controls (Horder et al., 2018). Importantly, they report no alterations of the GABA_A_ receptor or GABA_A_ α5 subunit availability in ASD model mice, such as the Shank3 knockout mouse and mice with a 16p11.2 deletion, consistent with the patient study.

## Discussion

The ASD mouse models, which are generated based on ASD risk genes, exhibit characteristic behavior specific to ASD. Additionally, altered neurotransmitter transporters and receptors are observed both in ASD mouse models and in patients with ASD, indicating that ASD mouse models are useful to investigate the biological mechanism of ASD. Although much evidence has been reported for functional and structural network changes in ASD mouse models and in patients with ASD, it is difficult to directly compare the results in mice and humans. How can we take the translational approach to the next step? Several issues should be addressed to link the research in a mouse model to clinical research. One of the significant problems is the anesthesia in mouse fMRI, even though the fMRI study uses light anesthesia. Given that mouse fMRI uses anesthetics for suppression of motion, it is difficult to directly compare the result of fMRI in the “unconsciousness” ASD mouse model with that in “consciousness” patients. At least, the effect of anesthesia on fMRI in mice should be investigated before discussing the result of mouse fMRI and clinical fMRI. In the previous study, we compared the resting-state functional connectivity between light anesthesia and awaked state (Tsurugizawa and Yoshimaru, 2021). Even low-dose anesthesia with isoflurane or medetomidine, commonly used in mouse fMRI, is found to inhibit functional connectivity more extensively than in the awake state. Additionally, weakened interhemispheric and subcortical connections are key connections under anesthetized conditions. Importantly, the typical structure of functional networks, such as the DMN and the sensory-motor network, are consistent between the lightly anesthetized state and awaked state. These results indicate that low-dose anesthesia can be useful to investigate the abnormality of the functional network in resting state, although the limitation of interpretation of fMRI under light anesthesia still exists. Despite common functional connectivity between awaked and lightly anesthetized state (Grandjean et al., 2020; Tsurugizawa and Yoshimaru, 2021), fMRI signal changes to the physiological stimuli are significantly disturbed by the anesthetic (Peeters et al., 2001; Tsurugizawa et al., 2010, 2016; Aksenov et al., 2015; Tokunaga et al., 2021; You et al., 2021). As an alternative, awake mouse fMRI is developed using habituation training to reduce stress during MRI experiments (Tsurugizawa et al., 2013, 2020, 2021; Bergmann et al., 2016; Yoshida et al., 2016; Madularu et al., 2017; Chen et al., 2020; Russo et al., 2021). Although the habituation protocol needs refinement to reduce stress efficiently (Low et al., 2016), awake fMRI can be an essential tool to bridge the human MRI and rodent MRI. Second, the phenotype of mouse models does not exactly match the symptoms of patients with ASD. This is important in the translational approach for the causality between altered gene, functional, and structural networks in ASD mouse models. To overcome this issue, the phenotype should be classified in animals. Previous studies report the phenotype and risk genes in ASD mouse models, which help in the planning of experiments using ASD mouse models (Silverman et al., 2010; Kazdoba et al., 2016a; Arakawa, 2020). However, some of the symptoms, e.g., sensory integration, of patients with ASD are not unknown in ASD mouse models. Third, the resting-state fMRI is performed in many ASD mouse model studies, but task-based fMRI, as well as DTI, was performed in a few studies. For task-based MRI, it may be due to the difficulty of task-based fMRI in mice with light anesthesia in addition to the limitation of the space in the MRI bore. In contrast, human fMRI reveals that patients with ASD show abnormalities in both the resting-state functional connectivity and the task-based fMRI response. Resting-state fMRI shows the functional separation of networks involved in sensation and cognition at the basal level. In contrast, task-based fMRI shows functional networks involved in specific task performance. Task-based fMRI may provide insight into functional abnormalities related to behavior and sensory processing specific to ASD.

In conclusion, accumulated evidence of comparable MRI data in ASD mouse models and in patients with ASD is helpful in the causal relationship between the ASD risk genes and abnormal brain function. In addition, a meta-analysis of MRI data using an open resource exchange platform will provide insights into consistent and distributed network abnormalities.

## Author Contributions

TT conceived, wrote, and prepared the review manuscript.

## Conflict of Interest

The author declares that the research was conducted in the absence of any commercial or financial relationships that could be construed as a potential conflict of interest.

## Publisher’s Note

All claims expressed in this article are solely those of the authors and do not necessarily represent those of their affiliated organizations, or those of the publisher, the editors and the reviewers. Any product that may be evaluated in this article, or claim that may be made by its manufacturer, is not guaranteed or endorsed by the publisher.
